# SGLT2 Inhibitors: The Next Blockbuster Multifaceted Drug?

**DOI:** 10.3390/medicina59020388

**Published:** 2023-02-16

**Authors:** Jonathan C. H. Chan, Michael C. Y. Chan

**Affiliations:** 1 Department of Medicine, Faculty of Medicine and Dentistry, University of Alberta, Edmonton, AB T6G 2R3, Canada; 2 Faculty of Pharmacy and Pharmaceutical Sciences, University of Alberta, Edmonton, AB T6G 2R3, Canada

**Keywords:** SGLT2 inhibitor, cardiorenal disease, cardiovascular disease, renal disease, diabetes, heart failure, cardiorenal protection

## Abstract

Sodium glucose cotransporter 2 inhibitor (SGLT2i) is a class of drugs that were originally intended for decreasing blood glucose in diabetes. However, recent trials have shown that there are other beneficial effects. Major clinical trials involving SGLT2i medications from 2015 to 2022 were reviewed using PUBMED search. Recent major SGLT2i landmark trials have demonstrated benefits for cardiovascular disease (reduce major adverse cardiovascular events (heart attack, stroke, cardiovascular death), hospitalization for heart failure, all-cause death), and renal disease (delay the onset of dialysis) regardless of diabetic status. The consistent cardiorenal benefits observed in major landmark trials have resulted in the rapid adoption of SGLT2i therapy not only in diabetes guidelines but also cardiovascular and renal guidelines.

## 1. Introduction

Diabetes, cardiovascular disease, and renal disease are all clinically related disease states. Diabetes is a major cause of chronic kidney disease and renal failure. Diabetes increases the risk for cardiovascular disease and death [1]. The top cause of death in diabetes is cardiovascular disease. In renal disease, a low estimated glomerular filtration rate (eGFR) is associated with higher mortality [2].

The origin of sodium glucose cotransporter 2 inhibitors (SGLT2i) is traced back to phlorizin, which is an organic compound first discovered and extracted from apple tree bark in 1835 by De Koninck and Stas [3]. It has played a role in diabetes research through its action of renal glucosuria and inhibition of glucose reabsorption. Originally intended for treating diabetes, SGLT2i has since intersected the fields of endocrinology, cardiology, and nephrology. In 2008, the United States Food and Drug Administration mandated the inclusion of cardiovascular outcomes in diabetes trials. Since this era of cardiovascular outcome trials, more benefits from SGLT2i have been discovered. The convergence of the treatment of diabetes, cardiovascular disease, and renal disease is a paradigm shift.

The objective of this review is to provide a summarized update on the recent major clinical outcome trials involving diabetes, cardiovascular disease, and renal disease populations. Within the last 10 years, many major cardiovascular and renal benefits have been discovered and clinical guidelines have subsequently reflected these benefits from SGLT2i therapy [4]. This review includes SGLT2i trials from 2015 to 2022.

## 2. Sodium Glucose Cotransporter 2 Inhibitor Trials in Type 2 Diabetes

The initial large clinical trials were focused on patients with type 2 diabetes, the main inclusion criterion.

The EMPA-REG OUTCOME trial was the earliest SGLT2i cardiovascular outcome trial and showed major cardiovascular benefits [5]. The trial studied 7020 patients with type 2 diabetes and patients received either empagliflozin or placebo. There was a reduction in major adverse cardiovascular events (MACE) (myocardial infarction, stroke, cardiovascular death) in the empagliflozin group (HR, 0.86; 95% CI, 0.74–0.99; *p* = 0.04 for superiority). Additionally, a reduction in hospitalization for heart failure was observed in patients receiving empagliflozin (HR, 0.65; 95% CI, 0.50–0.85; *p* = 0.002). This trial was the first positive cardiovascular outcome trial in type 2 diabetics.

The CANVAS Program trial compared canagliflozin to placebo in 10,142 patients with type 2 diabetes. A reduction in major adverse cardiovascular event was observed in the canagliflozin group (HR, 0.86; 95% CI, 0.75–0.97; *p* = 0.02 for superiority) [6,7]. Additionally, hospitalization for heart failure and cardiovascular death was reduced in the canagliflozin group (HR 0.78; 95% CI, 0.67–0.91). There was an increased risk of amputation, specifically at the toe or metatarsal in those that received canagliflozin.

The DECLARE-TIMI 58 trial evaluated dapagliflozin compared to placebo in 17,160 patients with type 2 diabetes [8,9]. There was a reduction in heart failure-related death and hospitalization (HR, 0.83; 95% CI, 0.73–0.95; *p* = 0.005). Notably, dapagliflozin did not reduce the rate of major adverse cardiovascular event (HR, 0.93; 95% CI, 0.84–1.03; *p* = 0.17). Renal events occurred less frequently in the dapagliflozin group (HR, 0.76; 95% CI, 0.67 to 0.87).

The TIMI Study Group performed a meta-analysis which included the EMPA-REG OUTCOME, CANVAS Program, and DECLARE-TIMI 58 trials with a total of 34,322 patients [10]. SGLT2i reduced hospitalization for heart failure (HR, 0.77; 95% CI 0.71–0.84; *p* < 0.0001) and progression of renal disease (HR, 0.55; 95% CI 0.48–0.64, *p* < 0.0001) in patients with or without cardiovascular disease or history of heart failure.

These trials showed the benefits of SGLT2i in reducing cardiovascular events in patients with type 2 diabetes (Table 1). This led to trials focusing on primarily examining cardiovascular benefits.

## 3. Sodium Glucose Cotransporter 2 Inhibitor Trials in Cardiovascular Disease

After observing such major cardiovascular benefits from SGLT2i in patients with type 2 diabetes, researchers decided to evaluate the potential cardiovascular benefits from SGLT2i therapy in patients without type 2 diabetes.

The DAPA-HF trial included 4744 heart failure reduced ejection fraction (HFrEF) patients receiving dapagliflozin versus placebo [13]. Dapagliflozin reduced the occurrence of the composite outcome of worsening heart failure or cardiovascular mortality (HR, 0.74; 95% CI 0.65–0.85; *p* < 0.001). Additionally, both hospitalization for heart failure (HR, 0.70; 95% CI 0.59–0.83) and cardiovascular mortality (HR, 0.82; 95% CI, 0.69–0.98) were reduced by dapagliflozin regardless of diabetic status.

The EMPEROR-Reduced trial compared empagliflozin to placebo in 3730 HFrEF patients [14]. The primary composite outcome of hospitalization for heart failure or cardiovascular death was reduced by empagliflozin (HR, 0.75; 95% CI, 0.65–0.86; *p* < 0.001). Empagliflozin reduced the number of hospitalizations for heart failure (HR, 0.70; 95% CI, 0.58–0.85; *p* < 0.001). The benefits of empagliflozin in reducing cardiovascular death and worsening heart failure was observed regardless of diabetic status.

The EMPEROR-Preserved trial compared empagliflozin to placebo in 5988 patients with heart failure with preserved ejection fraction (HFpEF) (ejection fraction above 40%) [15]. The primary outcome of hospitalization for heart failure or cardiovascular death was reduced by empagliflozin (HR, 0.79; 95% CI 0.69–0.90; *p* < 0.001) in both patients with or without diabetes. This result was mainly driven by the lowered risk of hospitalization for heart failure in those receiving empagliflozin.

The SOLOIST-WHF trial evaluated sotagliflozin and placebo in 1222 patients hospitalized for worsening heart failure, which included both HFrEF and HFpEF patients [16]. Interestingly, sotagliflozin is both a sodium glucose cotransporter 2 (SGLT2) inhibitor and sodium glucose cotransporter 1 (SGLT1) inhibitor. Sotagliflozin reduced cardiovascular death and hospitalization (HR, 0.67; 95% CI, 0.52–0.85; *p* < 0.001). This trial demonstrated that SGLT2i therapy can be started safely and effectively in patients even after an episode of decompensation [17]. Initiation of sotagliflozin before or after discharge significantly lowered cardiovascular death and urgent visits for heart failure.

The EMPULSE trial included 530 hospitalized patients with diagnosis of acute de novo or decompensated chronic heart failure regardless of left ventricular ejection fraction [18]. Patients were randomized to receive either empagliflozin or placebo. The primary outcome was a composite of death, number of heart failure events, time to first heart failure event, or a 5-point or greater change in the Kansas City Cardiomyopathy Questionnaire Total Symptom Score. Patients that received empagliflozin experienced greater benefit compared to the placebo group (stratified win ratio, 1.36; 95% CI, 1.09–1.68; *p* = 0.0054). The effectiveness of empagliflozin was observed in both acute de nove and decompensated chronic heart failure, regardless of ejection fraction or diabetic status. These clinical benefits could be observed in the 90 days after treatment initiation. The EMPULSE trial demonstrated that empagliflozin can be safely initiated in hospitalized patients for acute heart failure.

The DELIVER trial studied the role of dapagliflozin compared to placebo in 6263 heart failure patients [19]. This trial is the most inclusive heart failure trial. Namely, the trial included both hospitalized patients and outpatients with an ejection fraction of 40% or greater or an improved ejection fraction (previously EF < 40%). Dapagliflozin was shown to reduce the primary composite endpoint of cardiovascular death or worsening heart failure (HR, 0.82; 95% CI 0.73–0.92; *p* < 0.001). Those receiving dapagliflozin experienced lower total events and symptoms compared to the placebo group.

A pooled meta-analysis of the DAPA-HF and DELIVER trials demonstrated that dapagliflozin reduced the risk of cardiovascular death (HR, 0.86; 95% CI 0.76–0.97; *p* = 0.01), hospitalization for heart failure (RR, 0.71; 95% CI 0.65–0.78; *p* < 0.001), and major adverse cardiovascular event (HR, 0.90; 95% CI 0.81–1.00; *p* = 0.045), across a whole spectrum of left ventricular ejection fractions from ejection fraction of 25% to 65% [20]. This has widened the indications for SGLT2i.

These SGLT2i cardiovascular trials show the effective reduction of hospitalization for heart failure and cardiovascular death (Table 2). SGLT2i indications for cardiovascular disease continue to expand with subsequent cardiovascular outcome trial.

## 4. Sodium Glucose Cotransporter 2 Inhibitor Trials in Renal Disease

Renal benefits were signaled in various SGLT2i trials leading to the following trials examining the role of SGLT2i in renal disease.

The CREDENCE trial examined the use of canagliflozin and placebo in 4401 patients with type 2 diabetes and chronic kidney disease (CKD) [21]. The primary outcome of end-stage kidney disease, doubling of baseline serum creatinine, renal mortality, or mortality was reduced in patients on canagliflozin (HR, 0.70; 95% CI, 0.59–0.82; *p* = 0.00001). The secondary outcomes of cardiovascular death or hospitalization for heart failure were also reduced by canagliflozin (HR 0.69; 95% CI 0.57–0.83; *p* < 0.001). The CREDENCE trial showed that canagliflozin confers both cardiovascular and renal protection to patients with type 2 diabetes and chronic kidney disease patients. Adverse effect rates were comparable among the canagliflozin and placebo groups.

The DAPA-CKD trial studied 4304 patients with chronic kidney disease, regardless of diabetic status, receiving dapagliflozin [22]. The primary outcome (decline of estimated glomerular filtration rate (eGFR), new end-stage renal disease (ESRD), renal mortality, or cardiovascular mortality) was reduced in those receiving dapagliflozin (HR 0.61; 95% CI 0.51–0.72; *p* < 0.001). Additionally, dapagliflozin reduced hospitalization for heart failure and cardiovascular mortality (HR 0.71; 95% CI 0.55–0.92; *p* = 0.009). Importantly, the benefits of dapagliflozin extended to both patients with or without diabetes. Both dapagliflozin and placebo groups had a similar incidence of adverse effects.

The positive results from the CREDENCE and DAPA-CKD trials are timely since the RENAAL trial from two decades ago was the last positive trial involving chronic kidney disease patients [23].

The EMPA-KIDNEY trial studied 6609 patients chronic kidney disease with an estimated glomerular filtration rate between 20 and 45 mL/min per 1.73 m^2^ who received either empagliflozin or placebo [24]. The primary outcome of the progression of kidney disease (ESRD, decline in estimated glomerular filtration rate, or death from renal causes) or cardiovascular death was reduced in the empagliflozin group (HR 0.72; 95% CI 0.64–0.82; *p* < 0.001). Importantly, these results were consistent regardless of diabetic status. Aside from renal benefits, patients receiving empagliflozin had lower hospitalization than the placebo group (HR 0.86; 95% CI 0.78–0.95; *p* = 0.003).

The CREDENCE, DAPA-CKD, and EMPA-KIDNEY trials unequivocally show that SGLT2i dramatically delays the need for renal replacement therapies and reduces death from renal causes (Table 3).

## 5. Comparing SGLT2 Inhibitors and GLP-1 Receptor Agonists

With SGLT2i and GLP-1 receptor agonists having overlapping therapeutic profiles, the following subsections will explore key differences and situations to use one class more favorably or both.

### 5.1. SGLT2 Inhibitor Mechanism of Action

The primary mechanism of action of SGLT2i in glycemic control is the reduction of glucose reabsorption at the renal proximal tubule and thus glycosuria [25]. The mechanism of actions in SGLT2i in conferring cardiovascular and renal protection is still under investigation.

The proposed mechanisms of cardiovascular benefits include improved cardiac energetics, blood pressure reduction, inflammation reduction, naturesis/diuresis, inhibition of nervous system, and prevention of cardiac remodeling [26].

The proposed mechanisms of renal benefits include reduced intraglomerular pressure through increased sodium passing through the nephron leading to subsequent downstream effects of adenosine constricting afferent glomerular arterioles [27]. Improved tubular oxygenation and reduction in renal inflammation have also been proposed. Other mechanisms include the reduction of inflammatory markers (IL-6, TNF, IFNγ, NF-κβ, TLR-4, and TGF-β) and improved mitochondria function [28]. Work continues to be conducted to elucidate the mechanism of actions.

### 5.2. GLP-1 Receptor Agonist Mechanism of Action

Through the incretin effect, GLP-1 receptor agonists stimulate insulin release and inhibition of inappropriate glucagon secretion [29]. The bodyweight reductions are thought to be via the inhibition of gastric emptying and food intake.

The cardiovascular benefits of GLP-1 receptor agonists are proposed to be the result of reduction in inflammation [30], inhibition of platelet aggregation, and thrombus formation [31].

The renal benefits are suggested to be associated with reduced renal inflammation and structural preservation of renal function [32].

### 5.3. Bodyweight and HbA1c Reductions

The following trials compare the efficacy of SGLT2i with GLP-1 receptor agonists in reducing bodyweight and glycated hemoglobin (HbA1c).

The DURATION 8 trial compared dapagliflozin with exenatide in 695 patients with type 2 diabetes and poor glycemic control [33]. Patients received either dapagliflozin, exenatide, or a combination of dapagliflozin plus exenatide. Similar reductions in HbA1c were observed in patients that received dapagliflozin or exenatide alone, while dapagliflozin reduced bodyweight at a greater magnitude. Co-administration of both exenatide and dapagliflozin improved glycemic control and reduced cardiovascular risk factors. Combination therapy was well tolerated.

The SUSTAIN 8 trial compared subcutaneous semaglutide with canagliflozin in 788 individuals with uncontrolled type 2 diabetes [34]. Semaglutide showed superiority to canagliflozin in both bodyweight and HbA1c reduction.

The PIONEER 2 trial compared oral semaglutide with empagliflozin in patients with uncontrolled type 2 diabetes [35]. Semaglutide showed a greater HbA1c reduction but showed similar results in weight reduction. 

These three trials all show that GLP-1 receptor agonists are more effective than SGLT2i in simultaneously reducing bodyweight and HbA1c.

### 5.4. Major Adverse Cardiovascular Events

The following are observed in a meta-analysis of eight SGLT2i and GLP-1 receptor agonists trials. Both SGLT2i and GLP-1 receptor agonists reduced major adverse cardiovascular events (MACE). GLP-1 receptor agonists reduced the relative risk by 12% (HR, 0.88; 95% CI, 0.84–0.94; *p* < 0.001) and SGLT2i by 11% (HR, 0.89; 95% CI, 0.83–0.96; *p* = 0.001) [36].

For each component of major adverse cardiovascular event, SGLT2i had no effect on stroke while GLP-1 receptor agonists significantly reduced stroke (HR, 0.86; 95% CI, 0.77–0.97; *p* = 0.012). Both drug classes reduced cardiovascular death and myocardial infarction. GLP-1 receptor agonists have the benefit of the reduction of stroke events compared to SGLT2i.

### 5.5. Heart Failure

Only SGLT2i prevented hospitalization for heart failure [36]. With the pooled analysis of the DELIVER and DAPA-HF trials, SGLT2i shows benefit across all left ventricular ejection fractions. This is a benefit of SGLT2i compared to GLP-1 receptor agonists.

### 5.6. Renal Benefits

In a meta-analysis of both recent SGLT2i and GLP-1 receptor agonists landmark trials, both agent classes reduced the progression of renal disease; however, only SGLT2i prevented the delayed the decline of estimated glomerular filtration rate, renal death, and end-stage renal disease [36]. The DAPA-CKD, CREDENCE, and EMPA-KIDNEY trials support this [21,22,24]. These hard clinical outcomes show that SGLT2i have a greater benefit in patients with chronic kidney disease.

### 5.7. Combination Therapy of SGLT2 Inhibitors and GLP-1 Receptor Agonists

With the overlapping yet unique drug profiles, the combination of SGLT2i and GLP-1 receptor agonists may provide a synergistic effect in lowering HbA1c and preventing cardiovascular events. The following explores the use of combination therapy.

In a meta-analysis, combination therapy showed a greater reduction in HbA1c, bodyweight, and systolic blood pressure when compared to monotherapy [37]. In terms of adverse effects, hypoglycemia was increased from combination therapy. Other adverse effects included each respective medication’s adverse effect profile, as expected. This analysis demonstrated that combination therapy is safe and effective.

Deciding which agent to use depends on the patient comorbidities, patient preferences, and relates back to the mechanism of actions currently known and recent landmark trial results (Table 4). Further elucidation of cardiovascular and renal-related mechanism of actions and landmark trials will contribute to guiding therapy decisions.

The main differences between SGLT2i and GLP-1 receptor agonists are the following:

1. SGLT2i reduce hospitalization for heart failure;
2. GLP-1 receptor agonists reduce stroke;
3. GLP-1 receptor agonists are indicated in bodyweight loss;
4. SGLT2i are oral medications, while semaglutide is the only GLP-1 receptor agonist available in oral formulation.

## 6. Adverse Effects of SGLT2 Inhibitors

SGLT2i are safe medications with only mild and manageable adverse effects. The following subsections will introduce the most concerning adverse effects and the ways to mitigate these risks.

### 6.1. Urinary Tract Infections

In a meta-analyses of SGLT2i trials, there was no increased risk of urinary tract infections (UTI) from SGLT2i [38]. Intrinsically, patients with diabetes are at an increased risk of UTI [39]. Urinary tract infections are reliability treated with appropriate antibiotic medications.

### 6.2. Genital Mycotic Infection

SGLT2i increase the risk of genital mycotic infection, which is seen more commonly in females and uncircumcised males [40]. This is easily preventable through educating patients on the importance of daily hygiene practices. The treatment of genital mycotic infection includes the use of antifungal medications.

### 6.3. Volume Depletion

Since SGLT2i are mild diuretics, there is an increased risk of volume depletion-related adverse events such as hypotension, syncope, and dehydration [40]. Prior to initiating SGLT2i medications, the patient’s volume status should be evaluated. For example, dose adjustments on loop diuretics may be necessary in cases of low volume status.

### 6.4. Diabetic Ketoacidosis

During illness, patients with diabetes are at higher risk for diabetic ketoacidosis. As such, SGLT2i should be held when patients with diabetes experience illness and are at risk of dehydration [41].

### 6.5. Hypoglycemia

The risk of hypoglycemia from SGLT2i medications is very low. The risk is mainly increased in patients with diabetes taking concurrent hypoglycemics such as insulin or sulfonylurea. As such, these patients may need dosage adjustments when taking insulin or sulfonylureas [42].

### 6.6. Reduction in Estimated Glomerular Filtration Rate

It is observed that estimated glomerular filtration rate is reduced after the initiation of SGLT2i in the initial first to fourth week of initiation. This effect is transient and the estimated glomerular filtration rate is usually normalized by 1 to 3 months [43].

### 6.7. When to Hold SGLT2 Inhibitor

SGLT2i drugs should be held when patients are ill or unable to maintain adequate fluid intake or when undergoing major surgery or when the patient has an acute decline in renal function [4].

## 7. Initiating SGLT2 Inhibitors for Diabetes, Cardiovascular Disease, and Renal Disease

As previously discussed, SGLT2i are efficacious and safe medications with only mild and manageable adverse effects. The following discusses important points to keep in mind when initiating SGLT2i medications in patients.

The contraindications of SGLT2i include estimated glomerular filtration rate under 25 mL/min per 1.73 m^2^ and type 1 diabetes. Caution should be exercised when initiating SGLT2i in patients with volume depletion, active genital mycotic infections, hypotension under 95 mmHg, and diabetic ketoacidosis [44,45]. Patients should be warned to hold SGLT2i during illness and if they are unable to maintain fluid intake or acute kidney injury.

The following subsections will discuss the place of SGLT2i therapy in type 2 diabetes, cardiovascular disease, and renal disease.

### 7.1. SGLT2i for Type 2 Diabetes

Metformin is currently the first-line therapy in type 2 diabetes. Some have debated SGLT2i being first-line agents [46]. SGLT2i can be uptitrated after 4 to 12 weeks to reach glycemic target. If the patient has an HbA1c under 7.5% and is on insulin or sulfonylurea, a dose reduction can be considered, specifically a 10 to 20 percent dose reduction in insulin and a 50 percent dose reduction in sulfonylurea [47].

### 7.2. SGLT2i for Cardiovascular Disease

SGLT2i have now been adopted to be the first-line therapy in heart failure [4]. The pooled analysis of the DELIVER and DAPA-HF trials showed the ability to initiate SGLT2i at any left ventricular ejection fraction. After these trials, ejection fraction is becoming less relevant in terms of starting heart failure medications, especially SGLT2i medications. During the time of a patient’s hospitalization is a prime opportunity to initiate SGLT2i to prevent future complications [16,18].

### 7.3. SGLT2i for Renal Disease

In patients with both chronic kidney disease and type 2 diabetes with an estimated glomerular filtration rate equal to or over 30 mL/min per 1.73 m^2^, SGLT2i and metformin are recommended as first-line therapy [48]. The addition of SGLT2i is recommended even in patients meeting glycemic targets to prevent chronic kidney disease progression and cardiovascular disease. Patients should be evaluated and euvolemic before initiating.

## 8. Current Barriers to SGLT2 Inhibitor Initiation

Barriers to prescribing SGLT2i continues to be an important topic aside from the landmark trials. Despite the impressive benefits of SGLT2i and updated clinical guidelines, there remains a low uptake in prescribing SGLT2i for eligible patients [49]. This treatment inertia is a multifaceted challenge, including reasons such as the high price of SGLT2i medications, lack of insurance coverage, and unfamiliarity in medically complex patients [50].

Even though SGLT2i are safe medications, prescribers are more hesitant to prescribe these relatively newer medications and for newly approved indications. The reasons include misperception of adverse effects and limited guidelines in initiating SGLT2i in older patients [51]. Creation of SGLT2i initiation guidelines can help prescribers and increase usage of SGLT2i. Currently, of all prescribers, endocrinologists lead with the highest prescription of SGLT2i therapy [52,53]. Cardiologists tended to use SGLT2i over GLP-1 receptor agonists, with increased usage coinciding with the landmark cardiovascular outcome trials.

SGLT2i are expensive medications. Patients may only have access to SGLT2i if covered under insurance. However, formulary restrictions and insurance hurdles are an issue. Often, patients have to trial other classes of medications before being authorized to be eligible for SGLT2i. A cost-effectiveness study calculated that SGLT2i costs would have to decrease by at least 70% to be cost effective as first-line agents in type 2 diabetes [54]. Cost and insurance barriers are big factors that prevent patients from accessing these expensive yet efficacious and protective medications.

Hopefully, in the near future, reduced costs, education of prescribers, and improved financial coverage will lead to a greater uptake.

## 9. Discussion

Aside from the major outcome benefits in type 2 diabetes, various recent SGLT2i trials have consistently shown cardiovascular and renal benefits regardless of diabetic status [55]. There is strong and consistent evidence showing the benefits of SGLT2i medications in diabetes, cardiovascular disease, and renal disease. With these three diseases having overlapping pathology, SGLT2i are a unique treatment strategy to manage all three chronic diseases. SGLT2i medications are safe, with infrequent and manageable minor side effects such as genital mycotic infections [40].

New clinical guidelines have now adopted SGLT2i therapy after the release of the promising benefits of SGLT2i medications [4,56,57]. In type 2 diabetes, SGLT2i are second-line medications after metformin; however, recently, guidelines have started to recommend SGLT2i earlier in the course of treatment in high risk patients [58]. In heart failure, SGLT2i therapy is now first-line therapy in patients with or without diabetes [4]. With the recent DELIVER and DAPA-HF trials, SGLT2i are shown to be effective across all left ventricular ejection fractions. In renal disease and diabetes, SGLT2i and metformin are recommended as first-line therapy in patients with an estimated glomerular filtration rate equal to or over 30 mL/min per 1.73 m^2^ [48]. As additional landmark trials continue to be released, additional approved indications for SGLT2i should continue to arise.

Future research continues to examine the use of SGLT2i in type 1 diabetes and pre-diabetes. Currently, there are limited studies on SGLT2i use in type 1 diabetes [48]. Ongoing research continues to study the safety of SGLT2i use in estimated glomerular filtration rate under 30 mL/min per 1.73 m^2^. Future trials can further explore SGLT2i’s ability in lowering blood pressure. The EMPACT-MI trial is currently studying the use of empagliflozin in patients who had an acute myocardial infarction and examining the possibility in reducing the risk of heart failure and death.

The mechanism of actions pertaining to the cardiovascular benefits of SGLT2i is still under investigation. Proposed mechanisms include improved cardiac energy metabolism [26]. Continued research will help to better understand the impressive benefits of SGLT2i medications.

**Figure 1:**
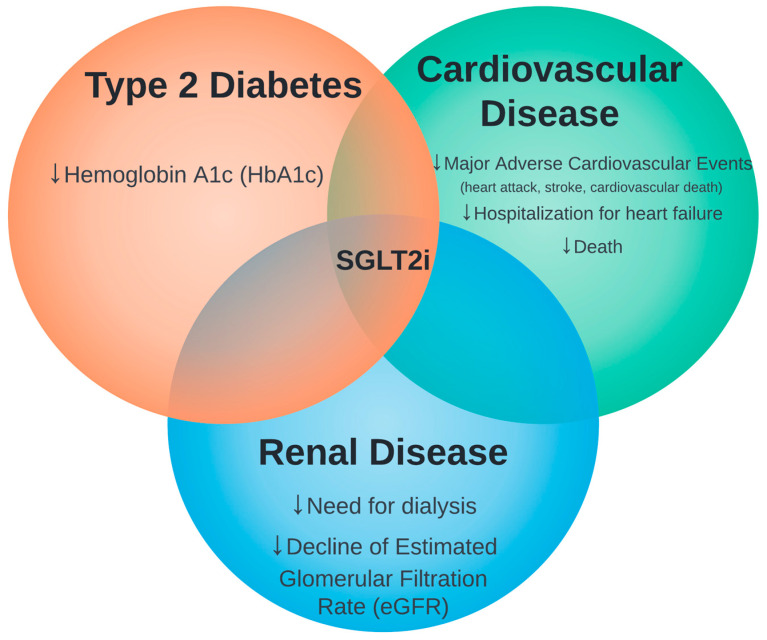
The intersection of sodium glucose cotransporter 2 inhibitors (SGLT2i) therapy in type 2 diabetes, cardiovascular disease, and renal disease.

Overall, SGLT2i consistently shows effectiveness in type 2 diabetes, heart failure, and chronic kidney disease and safety in various trials (Figure 1).

## 10. Conclusions

Major SGLT2i clinical trials have consistently shown both cardiovascular and renal benefits in addition to the glucose lowering effects of SGLT2i.

SGLT2i medications have shown major clinical benefits in three main patient groups:

1. Patients with type 2 diabetes;
2. Heart failure patients with any ejection fraction;
3. Patients with chronic kidney disease.

It has become clear in the last few years that SGLT2i is not merely a diabetic medication but actually a cardiovascular and renal disease-modifying agent. We think that SGLT2i are the blockbuster drugs of the early 21st century.

## Figures and Tables

**Table 1:** Sodium glucose cotransporter 2 inhibitors (SGLT2i) trials in type 2 diabetes.

Trial (Medication)	Main Outcome HR (95% CI) (*p*-Value)	Key Summary
EMPA-REG OUTCOME [5] (empagliflozin 10 or 25 mg)	↓ MACE, 0.86 (0.74–0.99) (*p* = 0.04) ↓ HHF ↓ All cause death	This was the first SGLT2i trial showing reduction of CV events.
CANVAS Program [6,11] (canagliflozin 100 or 300 mg)	↓ MACE 0.86 (0.75–0.97) (*p* = 0.02)	Canagliflozin reduced CV events and HHF.
DECLARE-TIMI 58 [8] (dapagliflozin 10 mg)	↓ CV death or HHF 0.83 (0.73–0.95) (*p* = 0.005)	Dapagliflozin reduced CV death and HHF. MACE was not reduced.
VERTIS CV [12] (ertugliflozin 5 or 15 mg)	MACE 0.97 (0.75–1.03) (*p* < 0.001 for noninferiority)	Ertugliflozin is non-inferior to placebo in reducing MACE.

CV, cardiovascular; eGFR, estimated glomerular filtration rate; HHF, heart failure for hospitalization; MACE, major adverse cardiovascular event.

**Table 2:** SGLT2i trials in cardiovascular disease.

Trial (Medication)	Main Outcome HR (95% CI) (*p*-Value)	Key Summary
DAPA-HF [13] (dapagliflozin 10 mg)	↓ composite of CV death and HHF 0.74 (0.65–0.85) (*p* < 0.001)	Dapagliflozin reduced the risk of worsening HF or CV death in HFrEF patients, regardless of diabetic status.
EMPEROR-Reduced [14] (empagliflozin 10 mg)	↓ composite of CV death and HHF 0.75 (0.65–0.86) (*p* < 0.001)	Empagliflozin shown to reduce HHF and CV death in HFrEF, regardless of diabetic status.
EMPEROR-Preserved [15] (empagliflozin 10 mg)	↓ CV death or HHF 0.79 (0.69–0.90) (*p* < 0.001)	Empagliflozin reduced CV death or HHF in HFpEF patients.
SOLOIST-WHF [16] (sotagliflozin 200 or 400 mg)	↓ CV death and HHF 0.67 (0.52–0.85) (*p* < 0.001)	This was the first major trial of SGLT1/SGLT2 inhibitor in hospitalized patients.
EMPULSE [18] (empagliflozin 10 mg)	↓Death, HF events, time to first HF event, ≥5 change in KCCQ score stratified win ratio, 1.36 (1.09–1.68) (*p* = 0.0054)	Empagliflozin is effective and can be safely initiated in hospitalized patients.
DELIVER [19]/Meta-analysis of DELIVER and DAPA-HF [20] (dapagliflozin 10 mg)	↓ CV death or worsening HF 0.82 (0.73–0.92) (*p* < 0.001)	Patients with HF with mildly reduced or preserved ejection fraction. Dapagliflozin benefits extend to all HF patients across a whole spectrum of EF.

CV, cardiovascular; EF, ejection fraction; HF, heart failure; HHF, hospitalization for heart failure; HFrEF, heart failure reduced ejection fraction; HFpEF, heart failure preserved ejection fraction; KCCQ, Kansas City Cardiomyopathy Questionnaire Total Symptom Socre.

**Table 3:** SGLT2i trials in renal disease.

Trial (Medication)	Main Outcome HR (95% CI) (*p*-Value)	Key Summary
CREDENCE [21] (canagliflozin 100 mg)	↓ ESRD, doubling of sCr, renal death, or CV death 0.70 (0.59–0.82) (*p* = 0.00001)	CREDENCE was the first trial in more than two decades in improving kidney endpoints.
DAPA-CKD [22] (dapagliflozin 10 mg)	↓ Decline in eGFR, new ESRD, renal death, or CV death 0.61 (0.51–0.72) (*p* < 0.001)	Dapagliflozin reduced the risk of eGFR decline, ESRD, and renal or CV death in CKD patients, regardless of diabetic status.
EMPA-KIDNEY [24] (empagliflozin 10 mg)	↓ ESRD, decrease in eGFR, renal death or CV death 0.72 (0.64–0.82) (*p* < 0.001) ↓ Hospitalization 0.86 (0.78–0.95) (*p* = 0.003)	Empagliflozin reduced ESRD, eGFR decline, and renal or CV death in CKD patients, regardless of diabetic status.

CKD, chronic kidney disease; CV, cardiovascular; eGFR, estimated glomerular filtration rate; ESRD, end-stage renal disease; GLD, glucose lowering drugs; sCr, serum creatinine.

**Table 4:** Comparing SGLT2 inhibitors and GLP-1 receptor agonists parameters.

Medication Parameters	SGLT2i	GLP-1 RA
Benefits	- Reduce MACE (but not stroke) - Reduce HHF - Reduce eGFR decline	- Reduce MACE - Weight loss
Routes of Administration	- Oral	- Injectable - Oral (semaglutide only)
Contraindications	- Type 1 Diabetes - eGFR <25 mL/min/1.73 m^2^	- Medullary thyroid carcinoma
Adverse Effects	- Genital mycotic infection	- Vomiting - GI upset
Rare Adverse Effects	- Euglycemic DKA	

CV, cardiovascular; DKA, diabetic ketoacidosis; eGFR, estimated glomerular filtration rate; GI, gastrointestinal; HHF, hospitalization for heart failure; MACE, major adverse cardiac event; UTI, urinary tract infection.

## Data Availability

Data sharing not applicable.
